# Functional Outcomes of Surgical Intervention for Tibial Plateau Fractures: A Study From a Tertiary Care Facility

**DOI:** 10.7759/cureus.92827

**Published:** 2025-09-21

**Authors:** Abdul Raoof, Jaisingh Rathod, Gavaskar Banothu, Satyanarayana Kummari, Mahipal R

**Affiliations:** 1 Orthopaedics and Traumatology, Kakatiya Medical College, Warangal, IND; 2 Orthopaedics and Traumatology, Area Hospital Nampally, Hyderabad, IND; 3 Orthopaedics and Traumatology, Rajiv Gandhi Institute of Medical Sciences Adilabad, Adilabad, IND; 4 Orthopaedics and Traumatology, General Hospital Nizamabad, Nizamabad, IND; 5 Radiodiagnosis, All India Institute of Medical Sciences, Bibinagar, Bibinagar, IND; 6 Radiodiagnosis, MNR Medical College and Hospital, Sangareddy, IND

**Keywords:** autogenous bone graft, falls from heights, intra-articular fractures, open reduction and internal fixation (orif), road traffic accident (rta), schatzker tibial plateau classification, tibial plateau fractures

## Abstract

Background

Among the most prevalent types of intra-articular fractures, tibial plateau fractures (TPFs) are common. These fractures can involve medial, lateral, or both plateaus and occur with varying degrees of articular depressions and displacements. The objective of this study was to examine the surgical treatment of TPFs to achieve a pain-free and flexible knee joint, avert the onset of osteoarthritis, and link radiological results with fracture type and functional outcomes.

Methodology

In this prospective, interventional study, 68 patients with TPFs were classified according to Schatzker’s classification system (SCS). The study was conducted for two years after obtaining approval from the Institutional Ethics Committee.

Results

TPFs were frequently observed in the active and productive demographic within our context. There was a disproportionate number of male patients with upper tibial fractures compared to female patients. There was a modest degree of right-sided preponderance in comparison to the left side. The majority of the patients (54/68 (79.4%)) were injured as a result of road traffic accidents (RTAs). The predominant fractures were classified as type I (22/68 (32.3%)) and type II (14/68 (20.5%)) according to SCS. Open reduction and internal fixation with a buttress plate and screws was used most frequently for TPFs. No instances of non-union were identified in the present investigation. The mean duration for union was 14 weeks, ranging from 10 to 22 weeks. Overall, 68 cases were treated with surgical procedures, and of those, 29 (42.6%) cases had great results, 33 (48.5%) cases had good results, 4 (5.8%) cases had fair results, and 2 (2.9%) cases had poor results.

Conclusions

It is beneficial to undergo surgical intervention to stabilize the knee when necessary, especially in cases of depressed and displaced fractures. Though challenging to treat surgically, TPFs can be successfully treated with stiff fixation and considerable anatomical reduction to restore articular congruity, increase early knee motion by minimizing post-traumatic osteoarthritis, and achieve optimal knee function.

## Introduction

Among the most prevalent types of intra-articular fractures (IAFs), tibial plateau fractures (TPFs) are rather common [1]. These fractures are the consequence of compressive forces that are either direct axial or indirect coronal in direction. Only 1% of all fractures are TPFs, although that number rises to 8% among the elderly [1]. Many different fracture patterns can occur with these fractures. There are different degrees of articular depressions and displacements associated with these fractures, which might affect the medial, lateral, or both plateaus. Each type of fracture exhibits distinct morphological characteristics and demonstrates a specific response to treatment. Considering that high-velocity trauma is associated with significant damage to both soft tissue and neurovascular tissue, it is crucial to ascertain the type of force that caused the injury. It is important to evaluate meniscal tears and ligament injuries in addition to TPF [2,3].

The prevalence of high-velocity injuries resulting from automotive accidents, along with the rise in overall road traffic incidents (RTAs), is generating a significant and escalating issue. As humans have begun to travel at high speeds while seated, with the load-bearing edge formed by bent hind limbs, a sudden stop of the vehicle results in the majority of the impact initially being absorbed by the patella, followed by the tibia and femur in differing ratios and positions [3-5].

The Schatzker classification system (SCS), which categorizes TPF into six types, is frequently utilized by orthopedic surgeons for the evaluation of the initial injury, care planning, and prognostic prediction. The severity of a fracture increases with each increasing numeric category, which indicates a greater energy level applied to the bone [3]. The initial four are unicondylar, while types V and VI are bicondylar. Each fracture type, as described by the SCS, provides orthopedic surgeons with the information they need to select the most effective treatment approach [4].

This study aimed to examine the surgical treatment of TPFs to achieve a pain-free and flexible knee joint, avert the onset of osteoarthritis, and link radiological results with fracture pattern and functional outcomes.

## Materials and methods

This prospective, interventional study included 68 patients with TPFs classified according to the SCS. The study was conducted for two years, from September 2016 to September 2018, after obtaining approval from the Institutional Ethics Committee, Kakatiya Medical College (approval number: IEC/KMC/2010-13/SPL). Informed written consent was obtained from each patient who participated in the research project.

Inclusion and exclusion criteria

The study included all patients, regardless of sex, who were above 18 years old and had a simple TPF according to SCS. The study excluded patients who were under the age of 18, had a compound TPF, were medically not suitable for surgery, or did not provide their consent to participate.

Preoperative instructions

A signed and informed consent from each patient was obtained before the administration of anesthesia and the surgical procedure. The patient was kept nothing by mouth for eight hours preceding surgery. Preoperatively, 0.5 cc of injection TT was administered intramuscularly immediately. Preoperative antibiotics were administered.

Surgical procedure (surgical approaches selected by fracture morphology)

Anterolateral Approach

The anterolateral approach was the most commonly employed technique for treating TPFs, as these injuries predominantly affect the lateral tibial plateau. This method is commonly employed for split-depression lateral plateau fractures (Schatzker type II) and bicondylar fractures (Schatzker types V and VI). Upon encountering an unstable tubercle fragment, an additional small anterior incision may be made for direct reduction and fixation of the tubercle.

Medial Approach

The medial approach was used for isolated fractures of the medial plateau (Schatzker type IV) and as part of the dual incision approach for bicondylar fractures. This method is suitable when the unstable fragment is located in the anterior aspect of the joint, and the fracture line runs parallel to the anteromedial surface of the tibia or is roughly aligned with the coronal plane.

Posteromedial Approach

This is an optimal method for standard shear fractures of the medial tibial plateau, particularly when the fracture line is situated in or near the coronal plane, necessitating buttress plating with fixation applied to the posterior or posteromedial surface of the medial tibial plateau. This method was employed for shear fractures where the fracture line is situated in or near the sagittal plane, necessitating buttress plating with fixation positioned on the medial surface (at the intersection of the posterior and anteromedial surfaces in the cross-section) of the tibial plateau.

Posterolateral Approach

The primary indication for using this method was a coronal fracture line that resulted in a displaced posterolateral fragment. This method should be limited to fracture patterns that cannot be managed by the anterolateral approach due to the potential for consequences, such as stiffness and peroneal nerve injury.

Direct Posterior Approach

The direct posterior approach should be restricted to fracture patterns that cannot be managed with posteromedial and posterolateral techniques due to the potential for iatrogenic damage to neurovascular structures in the popliteal fossa and the risk of flexion contracture. The injury pattern predominantly addressed with this method was a shear fracture of the posterior plateau, characterized by a principal fracture line in the coronal plane, or an avulsion of the PCL insertion accompanied by a fracture line extending into the extensive articular surface.

Reduction principles and techniques

Joint Exposure and Meniscal Management

A submeniscal arthrotomy or arthroscopy was employed to visualize the articular surface, evacuate hematoma, and protect/repair meniscal attachments where avulsed.

Elevation of the Depressed Articular Surface

Depressed fragments were elevated under direct and fluoroscopic guidance using a cortical window distal to the metaphysis or through metaphyseal tunnels. Tamponade with a tamp or bone impactor restored the subchondral arc.

Subchondral Support

The elevated surface was supported with subchondral rafting 3.5 mm screws placed parallel to the joint.

Buttress and Neutralization

Split fragments were reduced with pointed reduction forceps and provisionally fixed with K-wires. Definitive fixation utilized precontoured locking plates applied as buttress/antiglide constructs on the involved column(s).

Bone Void Management

Metaphyseal voids were filled with autologous iliac crest graft, allograft chips, or injectable calcium-phosphate substitute to prevent secondary collapse.

Alignment Restoration

Mechanical axis and tibial slope were checked clinically and with fluoroscopy; correction was confirmed on anteroposterior, lateral, and oblique views.

Postoperative care

Upon the successful attainment of rigid internal fixation, the patient was mobilized 48 hours after the removal of the catheters. For two to five days, the permitted range of motion was restricted to 0-20°. Commencing on the fifth day, the range of motion was progressively permitted to increase to 90° or beyond. Following the removal of sutures, a complete range of motion was permitted. In instances of uncertainty regarding the stability of fixation, external splinting via a plaster of Paris slab was employed for reinforcement. As part of the daily routine, range of motion exercises were performed under close observation, and the splint was reapplied. All patients were instructed and recommended to perform static quadriceps exercises as well as dynamic workouts using a quadriceps board as frequently as feasible and throughout the day. The recommended intervals between partial and full weight-bearing were 6-8 weeks and 12-16 weeks, respectively.

Postoperative instructions

Vital signs were monitored on an hourly basis. After the operation, analgesics were administered, and antibiotics were prescribed for 7-10 days. We continuously monitored the patients for any signs of bleeding. Because the surgeries were performed under spinal anesthesia, foot-end elevation was performed.

Statistical analysis

The information that was gathered was subsequently exported into Microsoft Excel version 2019 (Microsoft Corp., Redmond, WA, USA). SPSS Statistics for Windows version 22.0.0.0 (IBM Corp., Armonk, NY, USA) was utilized to perform the statistical analysis. Categorical variables were represented using numbers and percentages. On the other hand, quantitative variables were expressed by using the mean value in conjunction with the standard deviation (SD). Descriptive statistics were used to describe the study population. Inferential statistics were performed using the chi-square test in the case of qualitative variables and the Student’s unpaired t-test in the case of quantitative variables. Statistical significance was defined by a p-value <0.05.

## Results

TPFs are frequently observed in the active and productive demographic within our context, as individuals in this age group tend to participate in a greater number of activities and travel more frequently. The classification of fractures and their corresponding patterns is influenced by numerous factors, including the magnitude of the applied force, the age of the individual, the degree of knee flexion, the rate at which the force is applied, and the presence of valgus or varus stresses, among others.

In the current research, the predominant age groups among the patients were identified as 31 to 40 years, comprising 38 individuals, and 41 to 50 years, comprising 18 individuals. Regarding age, the youngest patient was 22 years old, and the oldest was 58 years old. High-velocity injuries were noted in younger patients, and low-velocity injuries were observed in older patients. No significant differences were observed in the functional outcomes between males and females. No significant association was observed between gender and functional outcomes (p = 0.79). There was a disproportionate number of male patients with upper tibial fractures compared to female patients. This was because, in the Indian setup, more women work indoors or in agriculture and rarely go outdoors (Table 1).

**Table 1:** Age and sex distribution.

	Number of cases	Percentage
Age (years)		
21–30	7	10.29
31–40	38	55.88
41–50	18	26.47
51–60	5	7.35
Total	68	100
Sex		
Male	61	89.70
Female	7	10.29
Total	68	100

There was a modest degree of right-sided preponderance in comparison to the left side. The majority of the patients who were treated in the current study were injured as a result of RTAs (54/68 (79.41%)), followed by injuries sustained as a result of falls from height (9/68 (13.23%)) (Table 2).

**Table 2:** Mode of injury.

Mode of injury	Number of cases	Percentage
Road traffic accident	54	79.41
Fall from height	9	13.23
Athletic injury	1	1.47
Assault	4	5.88
Total	68	100

The present investigation revealed that the predominant fractures were classified as type I (22/68 (32.35%)) and type II (14/68 (20.58%)) according to the SCS (Table 3).

**Table 3:** Different types of fractures.

Schatzker’s types	Type of fractures	Number of cases	Percentage
Type I	Pure cleavage	22	32.35
Type II	Cleavage with depression	14	20.58
Type III	Central depression	5	7.35
Type IV	Medial condyle Fracture	7	10.29
Type V	Bicondylar fracture	11	16.17
Type VI	Metaphysio diaphyseal dissociation	9	13.23
Total		68	100

**Figure 1:**
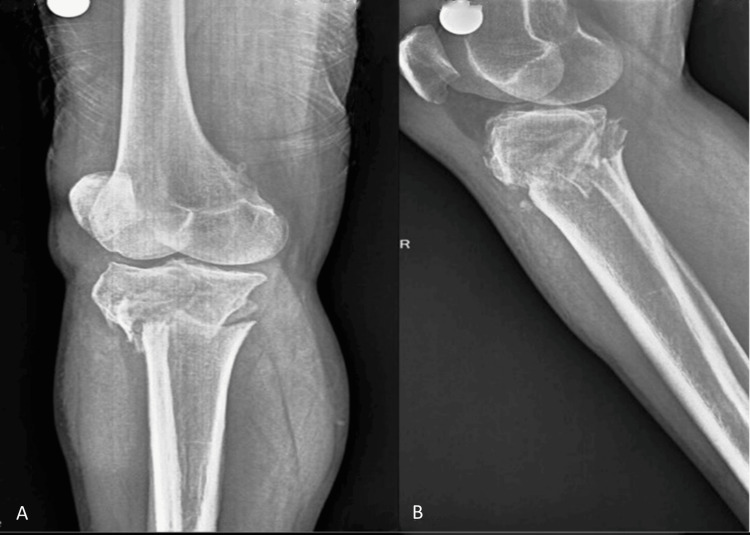
Preoperative radiographs of the right knee joint. (A) Anteroposterior and (B) lateral radiographs show comminuted intra-articular fracture of the medial and lateral tibial plateau with metadiaphyseal fracture.

**Figure 2:**
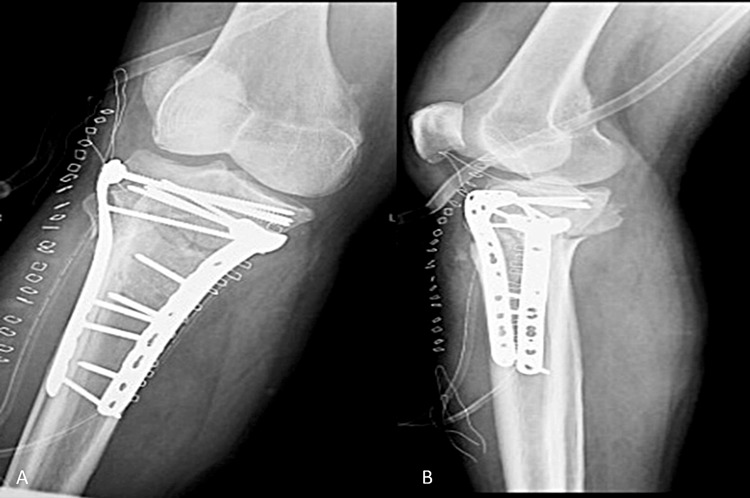
Postoperative radiographs of the right knee joint. (A) Anteroposterior and (B) lateral radiographs show the restoration of the articular surface and of the mechanical axis by open reduction and internal fixation.

In the present research, open reduction and internal fixation (ORIF) was the treatment that was utilized the most frequently for TPFss (Table 4; Figures 1, 2).

**Table 4:** Different methods of treatment. ORIF = open reduction and internal fixation

Method of treatment	Number of cases	Percentage
Percutaneous cancellous screw fixation	19	27.94
Cancellous screw and bone grafting	5	7.35
ORIF with buttress plate and screws	32	47.05
ORIF with buttress plate and bone graft	11	16.17
ORIF with buttress plate and external fixator	1	1.47
Total	68	100

When undergoing secure and rigid fixation, none of the patients were immobilized. In situations where there was uncertainty regarding the rigidity of the fixation, concomitant ligament injury, or osteoporosis, the immobilization, ideally in a cast above the knee, was extended for up to three weeks. There were three cases of infection and another two cases of severe metaphyseal comminution, both of which required immobilization for six to eight weeks.

Most cases had a satisfactory range of painless knee mobility (0-130°), except the final group, in which one patient experienced knee stiffness. Additionally, the three cases of wound infection were accompanied by stiffness in the knee joint.

All accompanying skeletal injuries were addressed, and the proper level of care was provided. Patients who suffer from ipsilateral shaft femur fractures demonstrate knee stiffness even after completing all follow-up procedures. After first being admitted to the Neuro-ICU under the supervision of neurosurgeons, the three patients who had presented with brain injuries underwent surgery for a tibial plateau fracture five to six days later. Although all of these concomitant fractures were present, the functional result of the TPF was not significantly affected.

All factures were united within the expected time frame. Not a single case of non-union was identified in the current study. The mean duration for union was 14 weeks, ranging from 10 to 22 weeks.

There were 68 cases that were treated with surgical procedures, and of those, 29 (42.6%) cases had great results, 33 (48.5%) cases had good results, 4 (5.8%) cases had fair results, and 2 (2.9%) cases had poor results. The severity of the injury and infections were the primary factors that led to adverse outcomes. A significant difference was observed in fracture type and functional outcomes. Low-velocity injuries (types I to III) had more favorable functional outcomes than high-velocity injuries (types IV to VI) (p = 0.03).

## Discussion

The TPF is one of the most prevalent types of IAFs. It is a serious traumatic injury that can be caused by RTAs, falls from heights, acts of aggression, and other similar incidents [1]. In certain cases, it is associated with other injuries that involve the bones or soft tissues. Fractures near the joint, particularly in the weight-bearing knee joint of the lower limb, are critically relevant because of their potential to cause considerable morbidity and reduce quality of life. Consequently, the management of TPF with intra-articular extension has emerged as a significant challenge for orthopedic surgeons [2-5].

In the current research, it was observed that most fractures occurred within the age range of 20 to 60 years, with the highest incidence observed in the age group of 31 to 40 years, accounting for 55.88%. Honkonen also reported an age occurrence of 20-60 years, with an average age of 39.8 years, which is consistent with the findings of the present investigation [6]. Additionally, Lee et al. found that patients with TPFs were 42 years old on average [7]. According to Albuquerque et al., 71% of injuries were noted in the 30-60-year age range, with the peak incidence seen in the 40-49-year age group [8]. In the current study, high-velocity injuries were noted in younger patients; nevertheless, low-velocity injuries were observed in older patients. In the study by Zeltser et al., the incidence of high-energy injuries was higher in younger patients, while the incidence of low-energy fractures was higher in older individuals [4].

A significant proportion of the patients in this study were males, accounting for 89.7% of the total. One possible explanation for this phenomenon is that the majority of the women in the Indian community are employed either indoors or in agriculture. Several investigations found that men were more impacted than females. These included Lee et al. (65.71%) [7], Albuquerque et al. (70.3%) [8], Manidakis et al. (58.4%) [9], and Mehin et al. (56%) [10]. In the current study, no significant differences were observed in the functional outcomes between males and females (p = 0.79).

In the present study, RTAs account for 79.41% of all injuries, followed by falls from heights (13.23%) and assaults (5.88%). On the other hand, the laterality of the fracture did not differ significantly. There were 61.7% of instances that impacted the right tibia, whereas 54.3% of the cases affected the left tibia.

Among the total fractures that were examined in the current research, Schatzker type I and type II fractures accounted for 52.93% of the total. The lateral condyle (Schatzker’s types I/II/III) was fractured in 64% of the patients, according to Rademakers et al. [11]. Mehin et al noted that roughly 30% of the injuries were high-grade type VI TPFs, while 35% were lower-grade type III fractures [10]. Based on MRI findings from 103 individuals, Gardner et al. [12] found that lateral plateau split-depression (Schatzker type II) fracture patterns were the most common.

This study examined 68 cases of uncomplicated TPF treated exclusively with surgical intervention. The criteria employed by various authors for the surgical treatment of these fractures varied. In the current investigation, a 3 mm depression was deemed a criterion for surgical intervention. Using a mean follow-up period of 28 months, Schatzkar reported 70 patients with TPFs of various sorts who were treated by conservative approaches (56% of cases) and surgical interventions (44% of cases). The conservative group had satisfactory outcomes in 58% of the cases, while open procedures achieved satisfactory outcomes in 78% of the cases [13]. During the initial half of the 20th century, two studies had attained a satisfactory proportion of favorable to outstanding short- and long-term outcomes with surgical intervention [14,15]. In another study, 159 cases of TPF of all patterns were examined, and the researchers found that surgical treatments produced better good-excellent results (84%) compared to conservative treatments (62%) [16]. Mehin et al. reported that 77% of the 286 patients who had TPFs received surgical treatment [10]. This was also documented by Pasa et al., where 30% of patients were treated conservatively, whereas 70% were treated surgically [17].

The strict requirements for a specific fixing technique for a given type of fracture have not been developed by us. This meant that each case was managed on an individual basis and in accordance with its requirements. The majority of type I, a small number of type II, and one type V case were fixed with percutaneous cancellous screws. ORIF was used to treat the split fracture, which had a displacement of more than 3 mm. Whenever it was deemed essential, bone grafting was incorporated into the ORIF procedure, along with the use of a buttress plate and screws in types II, III, V, and VI. Overall, 24 of the 68 patients who were diagnosed with TPF were treated with fixation using a cancellous screw and washer, while the rest of the patients were managed with a buttress plate (Figures 1, 2).

The advantages of initiating early knee motion encompass a reduction in knee stiffness and an enhancement in the healing (regeneration) of cartilage. Nonetheless, these advantages must be judiciously weighed against concerns, such as loss of fracture reduction, failure of internal fixation, and impaired healing of ligaments and soft tissues [18]. In their study, Schatzker et al. [13] found that the prognosis is determined by the degree of displacement, the type of fracture, the approach of treatment, and the quality of postoperative care. In the present investigation, we attained outstanding outcomes in 42.64% of patients and satisfactory results in 48.52% of patients, yielding an overall rate of 91.16% for acceptable outcomes, utilizing the conventional surgical treatment in conjunction with different established fixation techniques. Moreover, in terms of the functional outcome, we had 5.88% fair results and 2.94% poor results. These findings are consistent and comparable to those of previously published research in the field. In the current study, a significant difference was observed in fracture type and functional outcomes. Low-velocity injuries (types I to III) had more favorable functional outcomes than high-velocity injuries (types IV to VI) (p = 0.03).

Nevertheless, this study has some limitations. A small cohort of patients (n = 68) was selected from a single healthcare facility for the current study. This study was conducted over a relatively short period of time. Patients under 18 years of age, those with compound TPFs, and anyone deemed medically unfit for surgery were excluded from the study. The average follow-up duration of 48 months was insufficient to determine long-term outcomes and is unlikely to be adequate for the development of post-traumatic osteoarthritis. Logistical challenges hindered the recall of patients for objective clinical assessments of knee function, preventing us from establishing the association between functional outcome scores and actual clinical findings. Comprehensive research projects involving a larger number of participants can provide a more accurate portrayal of the subject under investigation.

## Conclusions

With the rise in the number of RTAs, there has been an increase in the number of TPFs, particularly those including high-velocity injuries. It is beneficial to undergo surgical intervention to stabilize the knee when necessary, especially in cases of depressed and displaced fractures. Though challenging to treat surgically, TPFs can be successfully treated with stiff fixation and considerable anatomical reduction to restore articular congruity, increase early knee motion, minimize post-traumatic osteoarthritis, and achieve optimal knee function.
